# Systematic Review and Meta-Analysis of Randomised Trials to Ascertain Fatal Gastrointestinal Bleeding Events Attributable to Preventive Low-Dose Aspirin: No Evidence of Increased Risk

**DOI:** 10.1371/journal.pone.0166166

**Published:** 2016-11-15

**Authors:** Peter C. Elwood, Gareth Morgan, Julieta Galante, John W. K. Chia, Sunil Dolwani, J. Michael Graziano, Mark Kelson, Angel Lanas, Marcus Longley, Ceri J. Phillips, Janet Pickering, Stephen E. Roberts, Swee S. Soon, Will Steward, Delyth Morris, Alison L. Weightman

**Affiliations:** 1 Cochrane Institute of Primary Care and Public Health, Cardiff University, Cardiff, United Kingdom; 2 Hywel Dda University Health Board, Llanelli, United Kingdom; 3 Department of Psychiatry, University of Cambridge, Cambridge, United Kingdom; 4 Division of Medical Oncology, National Cancer Centre, Singapore, Singapore; 5 Institute of Cancer & Genetics Cardiff University, Cardiff, United Kingdom; 6 Harvard Medical School, Boston, MA, United States of America; 7 University Clinic Hospital, University of Zaragoza, IIS Aragón, CIBEReshd, Zaragoza, Spain; 8 Welsh Institute for Health and Social Care, University of South Wales, Cardiff, United Kingdom; 9 Swansea Centre for Health Economics, Swansea University, Swansea, United Kingdom; 10 Medical School, Swansea University, Swansea, United Kingdom; 11 Department of Pharmacy, National University of Singapore, Singapore, Singapore; 12 Department of Cancer Studies, University of Leicester, Leicester, United Kingdom; 13 University Library Services, Cardiff University, Cardiff, United Kingdom; 14 Specialist Unit for Review Evidence (SURE), Cardiff University, Cardiff, United Kingdom; Kurume University School of Medicine, JAPAN

## Abstract

**Background:**

Aspirin has been shown to lower the incidence and the mortality of vascular disease and cancer but its wider adoption appears to be seriously impeded by concerns about gastrointestinal (GI) bleeding. Unlike heart attacks, stroke and cancer, GI bleeding is an acute event, usually followed by complete recovery. We propose therefore that a more appropriate evaluation of the risk-benefit balance would be based on *fatal* adverse events, rather than on the *incidence* of bleeding. We therefore present a literature search and meta-analysis to ascertain fatal events attributable to low-dose aspirin.

**Methods:**

In a systematic literature review we identified reports of randomised controlled trials of aspirin in which both total GI bleeding events and bleeds that led to death had been reported. Principal investigators of studies in which fatal events had not been adequately described were contacted via email and asked for further details. A meta-analyses was then performed to estimate the risk of fatal gastrointestinal bleeding attributable to low-dose aspirin.

**Results:**

Eleven randomised trials were identified in the literature search. In these the relative risk (RR) of ‘major’ incident GI bleeding in subjects who had been randomised to low-dose aspirin was 1.55 (95% CI 1.33, 1.83), and the risk of a bleed attributable to aspirin being fatal was 0.45 (95% CI 0.25, 0.80). In all the subjects randomised to aspirin, compared with those randomised not to receive aspirin, there was no significant increase in the risk of a fatal bleed (RR 0.77; 95% CI 0.41, 1.43).

**Conclusions:**

The majority of the adverse events caused by aspirin are GI bleeds, and there appears to be no valid evidence that the overall frequency of fatal GI bleeds is increased by aspirin. The substantive risk for prophylactic aspirin is therefore cerebral haemorrhage which can be fatal or severely disabling, with an estimated risk of one death and one disabling stroke for every 1,000 people taking aspirin for ten years. These adverse effects of aspirin should be weighed against the reductions in vascular disease and cancer.

## Introduction

Cardiovascular disease (CVD) and cancer are leading causes of death and disability in the world, with a combined treatment cost and global economic impact of approximately 2 trillion USD annually [1–5]. Low-dose aspirin (70–325 mg per day) is effective in the *secondary* prevention of vascular events in patients who have previously experienced heart attacks and strokes in [6,7,8], and more recently the US Preventative Services Task Force Agency has recommended low dose aspirin for the *primary* prevention of colorectal (CRC), and vascular disease in healthy individuals age 50 to 59, who have a 10-year CVD risk of 10% or greater.[9]

After a latency period of about five years, low-dose aspirin also appears to reduce the risk and mortality of colorectal and other cancers.[9–13] Furthermore, there is growing evidence that aspirin may be useful as an adjunct treatment in established colorectal and other cancers.[14]

The widespread adoption of aspirin for primary cancer and vascular prevention is however limited by concerns of toxicity, in particular major gastrointestinal (GI) bleeding, [8,9,15] ‘major’ being usually defined as bleeds that require blood transfusion together with those that lead to death. In a recent systematic overview aspirin was associated with a relative increase in GI risk (RR) of 1.4 (95% confidence intervals (CI) of 1.2–1.7), equivalent to an extra 0.5–3.6 bleeds per 1,000 person-years.[16] An increase in cerebral bleeding has also been attributed to low-dose aspirin, the RR estimated to be 1.4 (95% CI 1.2–1.7)[11] equivalent to one or two events per 10,000 subject-years.[17]

Nevertheless, low dose aspirin has been shown to have a favourable risk-benefit ratio when its cancer and vascular benefits are combined. Thun et al concluded that: ‘even a 10% reduction in overall cancer incidence [by aspirin] beginning during the first 10 years of treatment could tip the balance of benefits and risks favourably in average-risk populations’.[18] Pignone et al states: ‘aspirin appears beneficial for a large proportion of middle-aged men at low-moderate CHD risk’.[19] In a cautious meta-analysis Cuzick et al judged the benefit-harm balance to be favourable, with benefits increasing the longer aspirin is taken.[20] Two other evaluations which are limited to colorectal cancer and ignore the reduction in vascular disease also concluded that aspirin as a primary prevention strategy is cost-effective particularly when recommended alongside colorectal screening.[21,22] The most recent evaluation to be reported concludes that within the general population there would be benefit from aspirin use within the ages of 40–85 years.[23] In all these studies however, estimates of harm from aspirin are based on the *frequency* of ‘major’ GI together with cerebral bleeding.

Iatrogenic adverse events are problematic in healthy subjects, and especially in preventive interventions. Yet in the evaluation of the risk-benefit balance it is important to take account of the severity of the disease events prevented and caused by the intervention, and not just the frequencies of the events. Thus GI bleeds, which are sometimes severe, are acute events usually followed by recovery without sequelae, while strokes can leave residual physical and/or cognitive impairments in those who survive, and those who survive heart attacks or cancer may require complex and lifelong interventions.[24] It would therefore seem to be reasonable to consider fatal bleeds attributable to aspirin to be comparable in severity to the disease events prevented.

The distinction between GI bleeding and fatal bleeding is not trivial. A number of studies have already shown that GI bleeds attributable to aspirin carry a risk of death that is lower than that of spontaneous GI bleeding.[7,25–28].

If there is to be a genuine relationship of co-production in health promotion, the best available evidence should be shared with healthy subjects and with patients. Citizens involved in a ‘citizens’ jury’ have urged that the evidence relating to aspirin prophylaxis should be promoted and made easily available to the wider public [29] and it was this contact with the public that inspired this present research study.

The objective set for our study was a systematic review and meta-analysis of fatal GI bleeding attributable to low-dose aspirin. A specific aim was to estimate the frequency of fatal GI bleeds in subjects randomised to low-dose aspirin, relative to the fatality rate of spontaneous bleeds in those randomised not to take aspirin.

## Methods

### Literature searches

From pilot literature searches, using text words for aspirin and fatal bleeding, it became clear that a number of relevant studies known to the research team were not identified. This was because, although relevant information was contained in the body of some of the papers, words indicative of ***fatal*** bleeding were not included in the title or in the abstract.

A two part search was therefore developed and tested against known relevant papers, to ensure a highly sensitive search for publications with data on fatal bleeding events: (i) The subject search terms (for title, abstract and subject headings) were combined with a specific search developed to identify papers looking at aspirin adverse events. This enhanced search was run in Medline and Embase. The search combined subject headings (MeSH and Emtree as appropriate) for aspirin adverse event combined with (fatal* or death or mortality). (ii) A key word search to identify any mention of aspirin and fatal bleeding in the body of the text was conducted in Medline and CINAHL databases with full text capability using the terms (aspirin* or acetylsalicylic acid) and ((bleed* or bled* or haemorrhage or hemorrhage) and (fatal* or death or mortality)). Searches were completed in July 2016 and there were no date or language restrictions. Search strategies are summarised in S1 Table.

Given the challenges of finding papers with data on fatal bleeding events, supplementary search strategies were also employed. The reference lists of all included papers were examined in detail and emails requesting further data relevant to our purposes were requested from the authors when necessary.

Three of the authors (PCE, AW, DM) carried out the literature searching and identification of reports with relevant data. Papers were selected at title and abstract, and full text, stage by PCE and GM independently in duplicate. PCE extracted data from each report and the selection of data, and the correctness of every datum was checked by GM. An appraisal of bias in these studies [30] was conducted by ALW and DM independently in duplicate and is detailed in S2 Table. Discrepancies were resolved by discussion with PCE.

### Selection criteria

Studies were included in the main meta-analysis if they were randomised controlled trials of primary prevention or secondary prevention of further events, in which aspirin had been compared with a placebo; if ‘major’ gastrointestinal bleeding was reported; if the number of fatal GI bleeds was recorded or was ascertained either in correspondence with an author, or occasionally in another independent report. Intervention trials in general surgery, coronary artery procedures, stent insertion etc. in which aspirin had been randomised were excluded, being short-term, usually with multiple interventions additional to aspirin.

### Analysis plan

We analysed three outcomes: the risk of GI bleed attributable to aspirin, the risk of fatal GI bleed among those who bled, and the risk of a fatal bleed in subjects who had been randomised to receive aspirin. We used this last as a measure of risk of low-dose aspirin prophylaxis which is comparable to the disease risks reduced by the use of aspirin, namely a vascular event or a cancer. We also examined and comment upon estimates of fatal GI bleeding made by other authors, both in overviews and in observational studies.

### Statistics

Standard methods as recommended in Egger et al [31] were used throughout. Thus meta-analysis were conducted using random effects estimates, weighted by the method of DerSimonian and Laird,[32] with estimates of heterogeneity taken from the Mantel and Haenszel fixed effects model.[33] The summary statistic risk ratio (RR) was derived, and 95% confidence intervals estimated and heterogeneity assessed using the Q statistic. The random effects model was used throughout to incorporate an estimate of between study variation into the calculation of common effects. Begg’s funnel plots and Egger’s tests were examined as indicators of publication bias. Sensitivity analyses were conducted whereby each study was omitted in turn and the summary estimate recalculated to determine the influence of each study. All analyses were carried out using the statistical package STATA.

## Results

**Fig 1:**
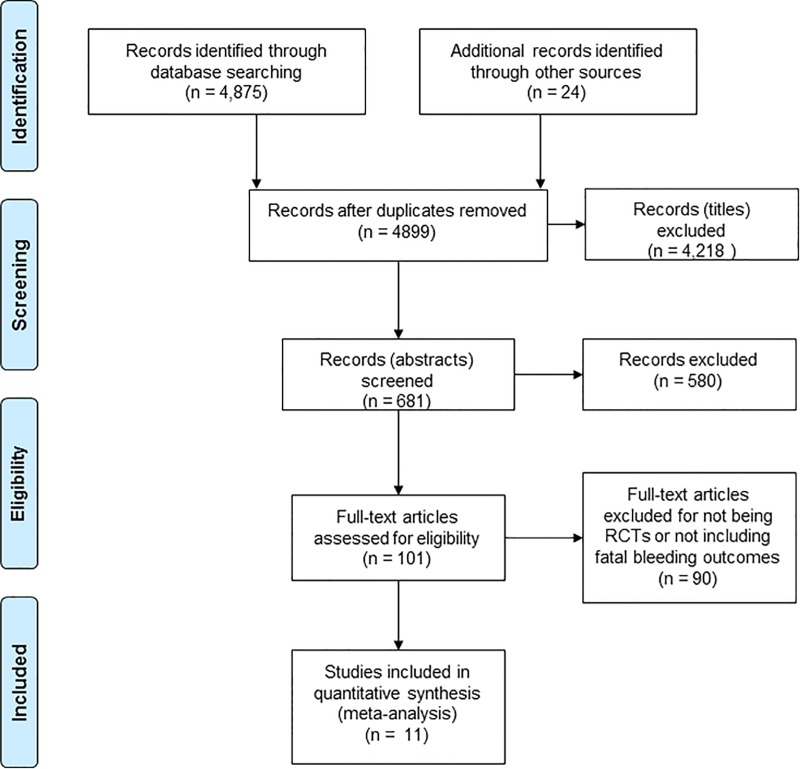
Flow diagram of the search and study selection.

A flow diagram of the search and study selection process can be seen in Fig 1. Authors of 38 papers were contacted by email to request further data. Eleven long-term randomised controlled trials of aspirin prophylaxis with data on fatal adverse effects were identified that met our selection criteria.[34–44]

Table 1 summarises data from these and S2 Table summarises aspects of the trials of relevance to quality and to possible bias. Nine trials were assessed as having low risk of bias [35–41,43,44] and two with unclear risk of bias. [34,42]

**Table 1:** Details of randomised trials.

Source		Dose of aspirin and duration of follow-up (range, mean or median)	Number of subjects		Number of bleeds		Fatal bleeds	
			Aspirin	No aspirin	Aspirin	No aspirin	Aspirin	No aspirin
Peto et al (1988)[34]	5,139 healthy male doctors	500mg daily or 300 enteric coated for 5–6 years	3,429	1,710	89	27	3	3
Physicians’ Health Study (1989)[35]	22,071 healthy physicians (United States)	325 mg alternate days for 60.2 months	11,037	11,034	13	6	1	0
Swedish Angina Trial (1992)[36]	1,360 patients with a transient ischaemic attack or myocardial infarction	75 mg daily for 32 months	676	684	9	4	1	1
Internat. Stroke Trial (1997)[37]	19,435 Patients with ischaemic stroke	300 mg daily for 6 months	4,858	4,860	23	14	4	2
Thrombosis Prevention trial (1998)[38]	5499 men at increased risk of vascular disease	75 mg daily for 6.8 years	1,268	1,272	7	4	1	2
Hansson et al (1998)[30]	Hypertensive patients on ‘optimal’ treatment	75 mg daily for 3.8 years	9,399	9,391	129	70	5	5
Primary Prev. Project (2001)[40]	4,495 selected from general practitioner lists	100 mg daily for 3–6 years	2,226	2,269	17	5	1	3
Baron et al (2003)[41]	1,121 patients selected at colo-rectal screening	81 mg daily 325 mg daily each for 3 years	377/372	372	2/4	3	0/1	0/0
Ridker (2005)[42]	39,876 women	100 mg alternate days for 10 years	19,934	19,942	127	91	2	3
Belch et al (2008)[43]	1,276 diabetic patients with arterial disease	1900 mg daily for 6.7 years	638	638	28	31	0	2
Brighton et al (2012)[44]	822 patients with venous thrombosis	100 mg daily for 37 months	411	411	8	6	0	2
**Risk of a GI bleed**								
	**On aspirin**	**8/1000**	**RR = 1.55** *(1*.*32*, *1*.*83) heterogeneity p = 0*.*401*					
	**On placebo**	**5/1000**						
**Risk of a bleed being fatal**								
	**On aspirin**	**4%**	**RR = 0.45** *(0*.*25*, *0*,*80) heterogeneity p = 0*.*783*					
	**On placebo**	**10%**						
**Risk of a fatal bleed if randomised to aspirin**								
	**On aspirin**	**3.7/10,000**	**RR = 0.77** *(0*.*41*, *1*.*43) heterogeneity p = 0*.*908*					
	**On placebo**	**4.7/10,000**						

Abbreviations: CI: confidence interval; mg: milligrams; RCT: randomised controlled trial; RR: risk ratio.

A meta-analysis gives no evidence of heterogeneity between the trials, and shows that aspirin increases the frequency of GI bleeds by about sixty percent, with a relative risk (RR) of 1.55 (95% confidence limits (CI) 1.32, 1.83; heterogeneity p = 0.401).

**Fig 2:**
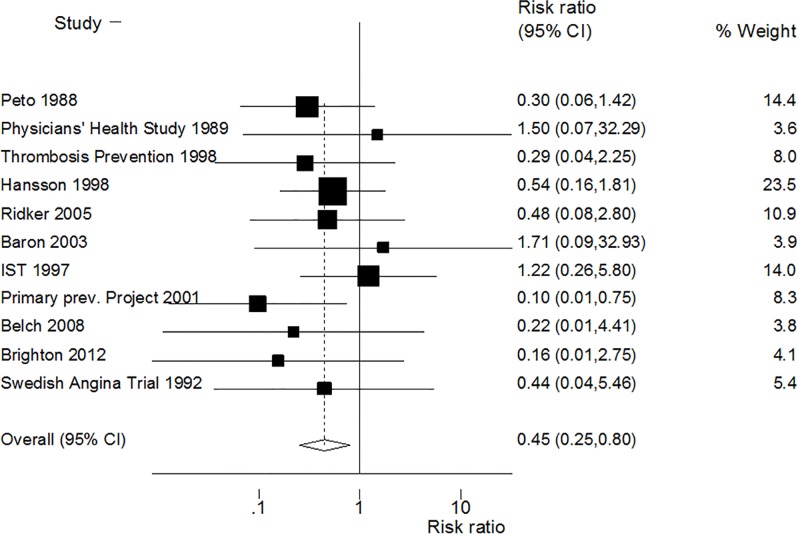
Forest plot of GI bleeds that led to death.

The forest plot in Fig 2 is based on data in Table 1 and shows that GI bleeds which led to death appear to be reduced in subjects who had been taking aspirin (RR 0.45; 95% CI 0.25, 0.80; heterogeneity p = 0.783). The sensitivity analysis (S1 Fig) shows that no individual study affected the overall RR dominantly. This procedure confirmed the stability of our overall result. We found no evidence of publication bias influencing this result (Egger’s test P = 0.754, see funnel plot in S2 Fig).

**Fig 3:**
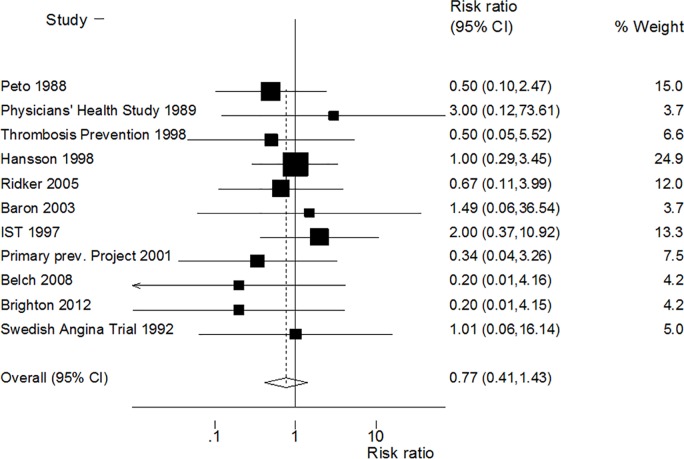
Forest plot of risk of subjects randomised to aspirin.

Of greatest relevance to the risk of prophylactic aspirin is however the risk of a fatal GI bleed in subjects who take low-dose aspirin, relative to the risk in those who take no aspirin. This risk was estimated in the randomised trials and is shown in a forest plot (Fig 3). There is no evidence of any significant increase in fatal GI bleeds attributable to aspirin. The risk of death from a bleed is: 3.7 ± 1.6 per 10,000 in subjects randomised to aspirin, and 4.7 ± 1.8 per 10,000 in subjects who had been randomised not to receive aspirin, the risk ratio (RR) being 0.77 (95% CI 0.41, 1.43 heterogeneity p = 0.908). The sensitivity analysis (S3 Fig) shows that, again, no individual study affected the overall RR dominantly, confirming the stability of the overall result. We found no evidence of publication bias influencing this result (Egger’s test P = 0.506, see funnel plot in S4 Fig).

## Discussion

Our meta-analysis, incorporating new data from direct email contact with the authors of some of the randomised aspirin studies, indicates that although aspirin increases the risk of GI bleeding by around 60%, low-dose aspirin is associated with a lower risk of fatality amongst patients who developed GI bleeding (RR 0.45; 95% CI 0.25, 0.80; heterogeneity 0.783). It is important to note that in no report was mention made of the use of gastric protection by a proton pump inhibitor (PPI) or other treatment as their use could have reduced some of the serious bleeding events caused by aspirin. Nor does our study take account of the use of enteric-coated aspirin, as this has not been shown to reduce risk of upper gastrointestinal complications.[45]

Furthermore, and most important, amongst the totality of all the subjects who had been randomised to take aspirin there was no increase in death from GI bleeding compared with subjects who had been randomised to take no aspirin (RR 0.77; 95% CI 0.41, 1.43; heterogeneity 0.908).

These findings are consistent with those from several earlier overviews of trials which have also suggested a reduction in the fatality of bleeds attributed to aspirin (Table 2). Data from these overviews were not included in our meta-analysis due to partial overlap with studies included in our analyses, together with the inclusion by these authors of some small and short term trials in patients undergoing surgery etc. These overviews all estimate the risk of death in subjects who bled, with the exception of the recent overview conducted for the U.S. Preventive Services Task Force in which fatal bleeds were examined within the totality of subjects,[9] and showed, as we have, no significant increase in fatal GI bleeds in subjects randomised to aspirin (OR 1.00; 95% CI 0.43, 2.36).

**Table 2:** Overviews of trials of GI bleeding and bleeds that were fatal in studies reported by other authors.

Source	Details	No. of subjects		No. of bleeds		No. of fatal bleeds		Risk of a GI bleed attributable to aspirin leading to death (95% CI)
		On aspirin	On placebo	On aspirin	On placebo	On aspirin	On placebo	
ATT (2009)[7]	6 RCTs	47,293	45,618	335	219	9	20	**OR 0.48** (0.17, 1.34)
Rothwell et al (2012)[25]	34 RCTs	40,269	40363	203	132	8	15	**OR 0.32** (0.12, 0.83)
Lanas et al (2011)[26]	28 RCTs	42,089^1^	42,089^1^	?	?	16	17	**OR 0.94** (0.47, 1.87)
McQuaid et al (2006)[27]	14 RCTs	25,964	25,993	48	28	Na^2^	Na^2^	**RR 1.23** (0.45, 3.41)
Wu et al (2016)[28]	12 RCTs	616	3,640	4^3^	18	0	3	**RR 0.29** (0.03, 1.22)
Present study	14 RCTs	56,654	55,016	468	285	24	29	**RR 0.45** (0.25, 0.80)

^1^ Only total numbers are given, equal numbers on aspirin and placebo assumed.

^2^ Na = Not available.

^3^ Estimated from data in the published paper.

Abbreviations: CI: confidence interval; OR: odds ratio; RCT: randomised controlled trial; RR: risk ratio.

Randomised trials are a valid source of evidence on these issues [19] and the above conclusions are therefore based entirely on randomised trials. Observational studies are prone to selection bias, are likely to be confounded, and polypharmacy is frequent. It is important however to consider how applicable our findings are likely to be to the broader community, since subjects involved in trials may have been selected to be at a low risk for GI bleeding, and subjects with gastric symptoms are likely to have been excluded.

Nevertheless, in an extensive review of observational community-based studies it was judged that: ‘The risks of major bleeding with low-dose aspirin in real-world settings are of a similar magnitude to those reported in randomised trials.[16] This confirms our own finding that the fatality rate of GI bleeds in our selected randomised trials (10.2%) and fatal bleeding rate (6.8%) in a selection of community based observational studies are reasonably similar.[46–53] In fact, it can very reasonably be argued on a practical level that clinical practice in the general community should be no less careful than selection of subjects for a trial.[24]

The strengths of our study lie in the fact that it is based on a systematic literature survey, supplemented with correspondence with 38 authors of studies of possible relevance. On the other hand, the number of available relevant data is small, and therefore the estimates we have made have a considerable degree of uncertainty. It should be noted however that the scarcity of fatal bleeding in contrast to the number of disease events prevented, is in itself an answer to the issue examined in our report.

The basic conclusion from our study is that although aspirin increases risk of GI bleeding, the overall risk of fatal bleeding is not significantly elevated, and the fatality rate, should GI bleeding occur, is significantly reduced.

How may these seemingly contradictory findings be reconciled? We suggest that the most likely explanation is that aspirin, through its anti-thrombotic effect may unmask gastrointestinal pathology early in its natural history, when the anatomical extent of the lesion is more limited, and the risk of massive uncontrolled bleeding lower and medical intervention is associated with the highest likelihood of success. A similar process may also occur with bleeding caused by Helicobacter pylori associated ulceration, or oesophageal varices.

A number of authors of observational studies of patients admitted with a GI bleed have recorded whether or not the patients had been taking aspirin, or were taking neither aspirin nor any other antiplatelet or anticoagulant drug. Thus Abu Daya et al commented that ‘Aspirin may have beneficial effects in patients with upper GI bleeding’.[51] Mose et al wrote: ‘low-dose aspirin… may even be associated with an improved outcome [in patients with peptic ulcer bleeding] compared with non-use’.[48] Ahsberg et al state ‘we found decreasing incidence … of non-variceal upper and lower GI bleeding during a period when prescription rates of drugs that increase the risk of GI bleed have increased several-fold. The increased use of these ‘high-risk drugs’ causes more severe bleeding but has had no influence on the risk of fatal outcome.’[49] Finally, Wehbeh et al gave their report the title: ‘Aspirin has a protective effect against adverse outcomes in patients with non-variceal upper gastrointestinal bleeding’.[50]

The main concern about intestinal bleeding focuses upon the upper GI tract. Bleeding also occurs from the lower intestinal tract, but the literature on this is sparse and most of the reports identified had been based on patients who had undergone colonoscopy, often with polypectomy, in whom large bowel disease appeared to account for the increase in lower GI bleeding attributable to aspirin.[54–57]

The few randomised studies which give evidence on lower GI bleeding from aspirin, show an increase, but the evidence is inconsistent,[16] and most of the evidence comes from the general population. A study conducted in Spain during 1996–2005 estimated the mortality of lower GI events to be 8.8%, compared to 5.5% for upper GI events.[58] Two further studies, also conducted in Spain, were based on the hospital records of 41 and 50 thousand patients admitted with a gastrointestinal event in 2001. In both the mortality of upper GI events was 5%, both in the total groups of patients and in the patients who had been taking aspirin or an NSAID.[46] Lower GI events accounted for 11–13% of the admissions, and 4–5% of these patients died. Amongst patients taking aspirin or an NSAID the mortality of lower GI bleeds was 4% in one of the studies and almost 12% in the other. A further study in Sweden examined the records of 731 patients admitted with non-variceal GI bleeding, and reported that the mortality of 440 patients with upper GI bleeding, and 289 with lower GI bleeding was 3% in both.[59]

These reports do not give adequate evidence on the relevance of aspirin to the bleeding reported and we therefore feel that the evidence on lower GI bleeding is too limited and too inconsistent to support any reliable conclusion relevant to the issues examined in our report.

Unlike a gastrointestinal bleed, the consequences of a cerebral bleed, whether or not fatal, are of a severity comparable to the effects of a myocardial infarct or a cancer. Mortality is around 30–50% [7,60–62] and severe physical and psychological disablement are possible in those who survive.[17] It would therefore seem to be reasonable to include all cerebral bleeds, fatal and non-fatal, in an evaluation of the risk-benefit balance of aspirin prophylaxis.

Fortunately cerebral bleeds attributable to aspirin are rare. The risk ratio associated with aspirin is about 1.4 (95% CI 1.2–1.7),[16] equivalent to one or two haemorrhagic strokes per year in every 10.000 subjects on aspirin.[17] Gorlick & Wiseman however comment on the probable reduction in cerebral bleeding if blood pressure is measured before aspirin prophylaxis is started, and hypertension, if present, is adequately treated.[17] Evidence to support this comes from a trial based on more than 18,000 hypertensive patients, all of whom were receiving optimal antihypertensive treatment. Amongst almost ten thousand patients randomised to aspirin there seven fatal bleeds and eight (P<0.001) in those on placebo.[39]

An important issue in the evaluation of prophylactic aspirin is the duration of aspirin taking as this is relevant both to the benefits, and to the risks of the drug. While reductions in vascular disease appear to commence immediately, almost all studies of cancer prevention show a 3-5-year delay before benefit from aspirin is clinically apparent, and thereafter the reduction increases.[25,63] This delay had been predicted on the grounds that prevention by aspirin occurs at a cellular level and there is an inevitable delay before an effect upon a cell becomes apparent in the absence of a tumour.[64]

On the other hand, the incidence of GI bleeding attributable to aspirin appears to decrease over time. Within the first month of aspirin taking the risk is increased more than four-fold,[63,65] but it then reduces rapidly and after about three to five years of aspirin taking there appears to be no excess in GI bleeds (OR 0.63; 95% CI 0.34, 1.16).[25] Likewise, most of the deaths from bleeding occur within the first month of aspirin taking,[66] implying the presence of underlying, untreated gastric pathology.

Additional to these changes, reports from a number of countries indicate that gastrointestinal bleeding and fatal bleeding have decreased substantially over time, probably because of better patient care, increased use of gastroprotective drugs, lower doses of aspirin and less use of other NSAIDs, and more effective treatment of the bleeding. These reductions have been large: almost 50% in Scotland over an 18 year period,[67] to almost half in Spain over ten years,[58] by almost 20% over 18 years in the USA,[68] and over 25% within seven years in Wales.[69]

## Conclusions

Gastrointestinal bleeds constitute the majority of the adverse events caused by aspirin. The increase is about 60% overall, but there appears to be no increase in fatal GI bleeds attributable to low-dose aspirin, indeed prophylactic aspirin appear to be associated with a reduction in the fatality of GI bleeds.

The undesirable effect of prophylactic aspirin which is of a severity comparable to a vascular disease event or a cancer is a bleed that leads to death, and low-dose aspirin appears to be associated with one death and one disabling haemorrhagic stroke per year in every 10,000 people taking low-dose aspirin. The available evidence makes it seems likely that these cerebral events would be reduced if hypertension is identified and adequately treated.[7,17,39]

In addition, there will be one or two non-fatal GI bleeds per 1,000 people each year, but the frequency of these bleeds appears to fall rapidly, and there is no evidence of any increase in GI bleeds attributable to aspirin after three or four years of prophylaxis

All these conclusions are relevant to the risk-benefit balance of aspirin prophylaxis and should be communicated to subjects at risk of vascular disease and/or cancer, to enable them to make an informed decision about the protection of their own health.[29,70]

## Supporting Information

S1 Fig Sensitivity analysis repeating meta-analysis in Fig 2 in the text, excluding one study at a time. The middle vertical axis indicates the overall RR and the two vertical axes indicate its 95% CI. Every hollow circle indicates the pooled RR when the left study was omitted in this meta-analysis. The two ends of every broken line represent the 95% CIs.
(TIF)

S2 Fig Begg’s funnel plot with estimated 95% confidence intervals for meta-analysis in Fig 2 in the text. (TIF)

S3 Fig Sensitivity analysis repeating meta-analysis in Fig 3 in the text, excluding one study at a time. The middle vertical axis indicates the overall RR and the two vertical axes indicate its 95% CI. Every hollow circle indicates the pooled RR when the left study was omitted in this meta-analysis. The two ends of every broken line represent the 95% CIs.
(TIF)

S4 Fig Begg’s funnel plot with estimated 95% confidence intervals for meta-analysis in Fig 3 in the text. (TIF)

S1 Table Literature search on 25 August 2016. No date restrictions, no language restrictions. (DOCX)

S2 Table Results of the Cochrane risk of bias assessment. Based on: Cochrane Handbook for Systematic Reviews of Interventions. [28]
(DOCX)
