# Supplementary figures and images for: Systematic Review and Meta-Analysis of Randomised Trials to Ascertain Fatal Gastrointestinal Bleeding Events Attributable to Preventive Low-Dose Aspirin: No Evidence of Increased Risk
Source: PLoS One. 2016 Nov 15;11(11):e0166166. doi: 10.1371/journal.pone.0166166 (PMC5113022; doi:10.1371/journal.pone.0166166)

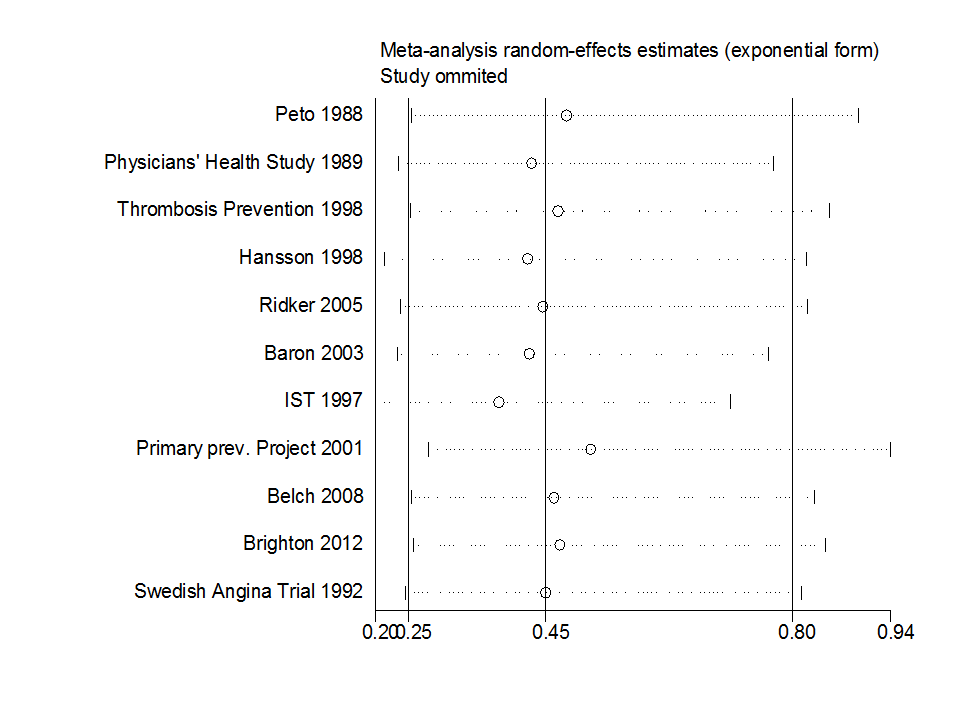

Supplement: S1 Fig — The middle vertical axis indicates the overall RR and the two vertical axes indicate its 95% CI. Every hollow circle indicates the pooled RR when the left study was omitted in this meta-analysis. The two ends of every broken line represent the 95% CIs. (TIF) [file pone.0166166.s001.tif]

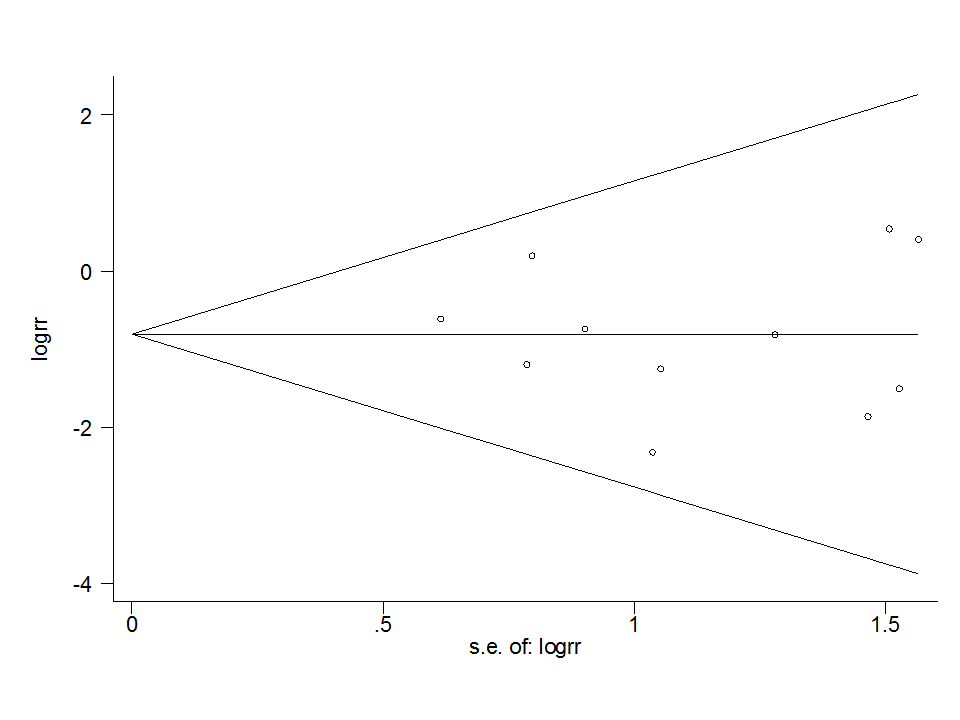

Supplement: S2 Fig — (TIF) [file pone.0166166.s002.tif]

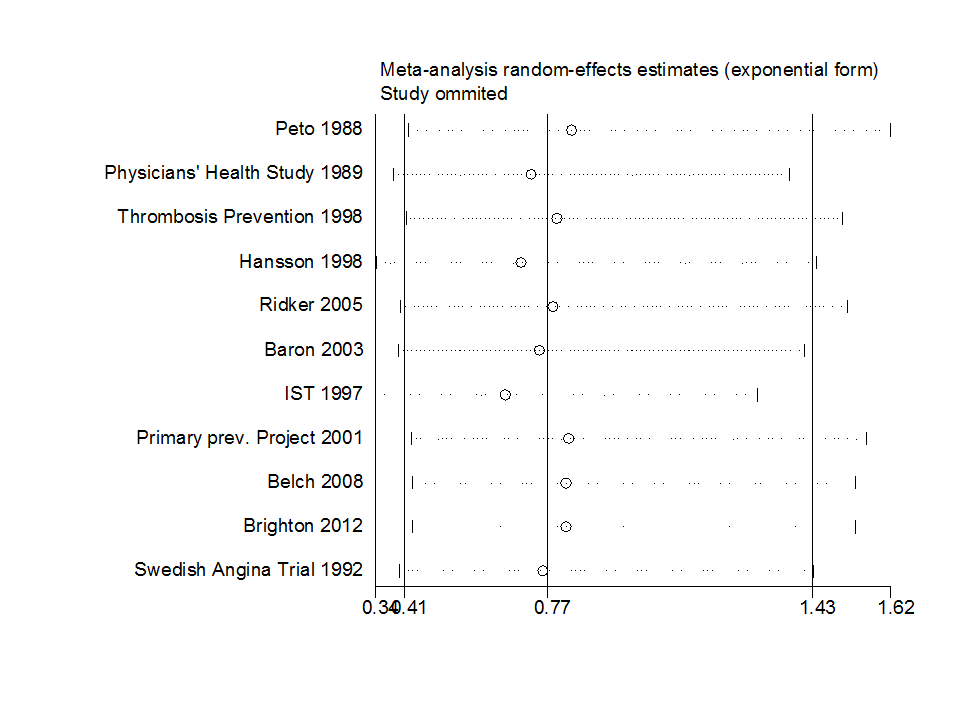

Supplement: S3 Fig — The middle vertical axis indicates the overall RR and the two vertical axes indicate its 95% CI. Every hollow circle indicates the pooled RR when the left study was omitted in this meta-analysis. The two ends of every broken line represent the 95% CIs. (TIF) [file pone.0166166.s003.tif]

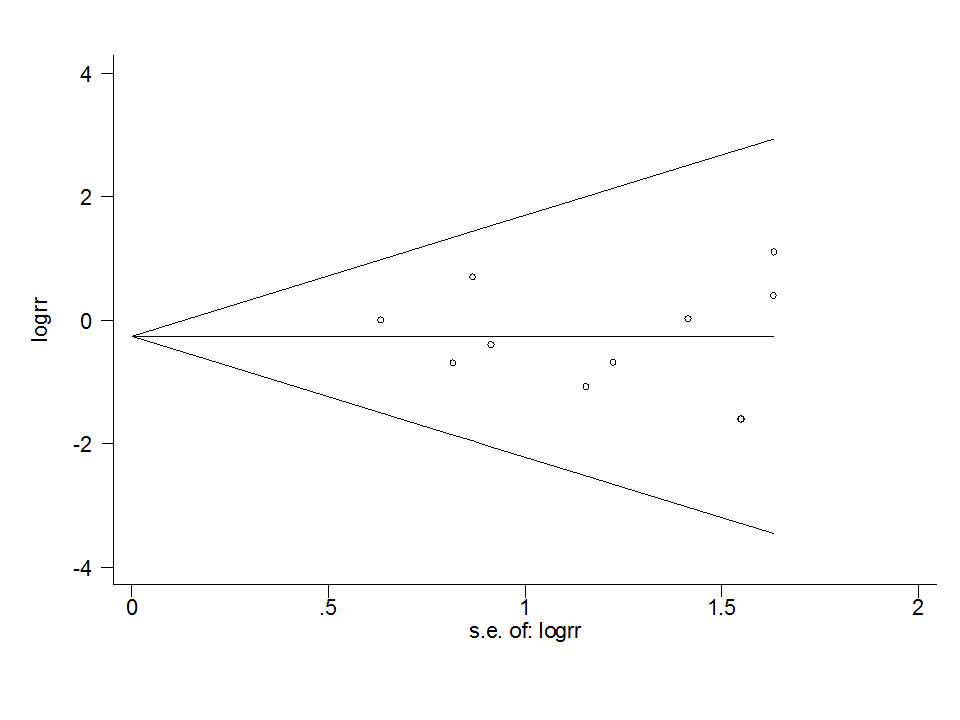

Supplement: S4 Fig — (TIF) [file pone.0166166.s004.tif]
