# Supplementary material for: Systematic Review and Meta-Analysis of Randomised Trials to Ascertain Fatal Gastrointestinal Bleeding Events Attributable to Preventive Low-Dose Aspirin: No Evidence of Increased Risk
Source: PLoS One. 2016 Nov 15;11(11):e0166166. doi: 10.1371/journal.pone.0166166 (PMC5113022; doi:10.1371/journal.pone.0166166)
Supplement: S1 Table — (DOCX) [file pone.0166166.s005.docx]

S1 Table. Literature search on 25 August 2016. No date restrictions, no language restrictions.

**Literature search part (i): Aspirin adverse events**

**Medline [OVID] 1946-:**

1. exp Aspirin/ae [Adverse Effects]

2. aspirin*.tw.

3. acetylsalicylic acid*.tw.

4. 2 or 3

5. (ae or to or po or co).fs.

6. (safe or safety).ti,ab.

7. side effect$.ti,ab.

8. ((adverse or undesirable or harms$ or serious or toxic) adj3 (effect$ or reaction$ or event$ or outcome$)).ti,ab.

9. exp product surveillance, postmarketing/

10. exp adverse drug reaction reporting systems/

11. exp clinical trials, phase iv/

12. exp poisoning/

13. exp substance-related disorders/

14. exp drug toxicity/

15. exp abnormalities, drug induced/

16. exp drug monitoring/

17. exp drug hypersensitivity/

18. (toxicity or complication$ or noxious or tolerability).ti,ab.

19. exp Postoperative Complications/

20. exp Intraoperative Complications/

21. or/5-20

22. 4 and 21

23. 1 or 22

24. exp Gastrointestinal Hemorrhage/

25. (gastrointestin* adj3 (hemorrhage* or haemorrhage* or bleed* or bled*)).tw.

26. exp Cerebral Hemorrhage/

27. ((cerebral or cerebrum) adj3 (hemorrhage* or haemorrhage* or bleed* or bled*)).tw.

28. or/24-27

29. (fatal* or death or mortalit*).tw.

30. exp Death/

31. exp Mortality/

32. 29 or 30 or 31

33. 23 and 28

34. 32 and 33

**Embase [OVID] 1947-:**

1. exp acetylsalicylic acid/ae [Adverse Drug Reaction]

2. aspirin*.tw.

3. acetylsalicylic acid*.tw.

4. 2 or 3

5. (ae or si or to or co).fs.

6. (safe or safety).ti,ab.

7. side effect$.ti,ab.

8. ((adverse or undesirable or harm$ or serious or toxic) adj3 (effect$ or reaction$ or event$ or outcome$)).ti,ab.

9. exp adverse drug reaction/

10. exp drug toxicity/

11. exp intoxication/

12. exp drug safety/

13. exp drug monitoring/

14. exp drug hypersensitivity/

15. exp postmarketing surveillance/

16. exp drug surveillance program/

17. exp phase iv clinical trial/

18. (toxicity or complication$ or noxious or tolerability).ti,ab.

19. exp postoperative complication/

20. exp Peroperative Complication/

21. or/5-20

22. 4 and 21

23. 1 or 22

24. exp gastrointestinal hemorrhage/

25. (gastrointestin* adj3 (hemorrhage* or haemorrhage* or bleed* or bled*)).tw.

26. ((cerebral or cerebrum) adj3 (hemorrhage* or haemorrhage* or bleed* or bled*)).tw.

27. or/24-26

28. (fatal* or death or mortalit*).tw.

29. exp death/

30. exp mortality/

31. or/28-30

32. 23 and 27 and 31

**Literature search part (ii): Full text search for aspirin and fatal bleeding**

**Medline Complete [EBSCOhost] 1865-**

**CINAHL Complete [EBSCOhost] 1813-**

[Aspirin](https://ovidsp.uk.ovid.com/sp-3.16.0b/ovidweb.cgi?&S=AKHBPDGGJOHFKCFJFNKKBCOFBBCEAA00&Search+Link=%2a%22Aspirin%22%2f) [MH Exact Subject Heading] and (fatal* or death or mortality)) [TX All Text]
OR
(aspirin* or acetylsalicylic acid) [TX All Text] and ((bleed* or bled or haemorrhage or hemorrhage) and (fatal* or death or mortality)) [TX All Text]
