# Supplementary material for: Systematic Review and Meta-Analysis of Randomised Trials to Ascertain Fatal Gastrointestinal Bleeding Events Attributable to Preventive Low-Dose Aspirin: No Evidence of Increased Risk
Source: PLoS One. 2016 Nov 15;11(11):e0166166. doi: 10.1371/journal.pone.0166166 (PMC5113022; doi:10.1371/journal.pone.0166166)
Supplement: S2 Table — [28] (DOCX) [file pone.0166166.s006.docx]

S2 Table. Results of the Cochrane risk of bias assessment. Based on: Cochrane Handbook for Systematic Reviews of Interventions.[28]

| **First author, date** | **Sequence generation^1^** | **Allocation concealment** | **Blinding^2^** | **Complete outcome^3^** | **No selective reporting** | **Lack of other bias^4^** | **Overall risk** | **Notes** |
| --- | --- | --- | --- | --- | --- | --- | --- | --- |
| Anon. 1998 [36] | Yes | Yes | Yes | Yes | Yes | Yes | Low | 98.9% follow up. High compliance with treatment (98%) initially though reduced to 14% at 1 year, 29% at 3 and 42% at 5 years. Overall ~80% assumed. |
| Baron et al. 2003 [39] | Yes | Yes | Yes | Yes | Yes | Yes | Low | 96.7% follow-up and 94% compliance to ca 3 years. |
| Belch et al.2008 [41] | Yes | Yes | Yes | Yes | Yes | Yes | Low | Not clear if ITT analysis but 84% follow up. 86% compliance to 12 months, 50% to 5 years. Overall ~80% compliance assumed. |
| Brighton 2012 [42] | Yes | Yes | Yes | Yes | Yes | Yes | Low | ITT analysis. 15.1% of C and 11.9% of I group discontinued medication over the follow up period (up to 4 years). Overall compliance >80% |
| Collab Group 2001 [38] | Yes | Yes | No | Yes | Yes | Yes | Low | Participants were not blinded to treatment but would not influence bleeding outcomes. Outcome assessment was blinded. Protocol not published but approved by commitee and outcomes pre-specified. 92.3% follow up. At year 1 and at end of the study (mean follow up 3.6 years) 80.8 and 80.7% compliance with aspirin therapy. |
| Hansson et al 1998 [37] | Yes | Yes | Yes | Yes | Yes | Don’t know | Low | Multi-country trial and protocol effectively approved in advance. Double blinded with respect to aspirin treatment (though open re hypertension treatment). 97.4% follow up - average length 3.8 years. Compliance with therapy is not reported but a huge well conducted study so overall risk of bias proposed as low. |
| Peto et al 1988 [32] | Yes | Don't know | No | Yes | Don't know | Yes | Unclear | Baseline characteristics evenly balanced other than pre-randomisation systolic blood pressure (1 mm Hg higher in the aspirin group). Participants were not blinded to treatment but would not influence bleeding outcomes. Outcomes were blinded. ITT analysis but some in the control group had started taking aspirin during trial. 99% outcome data for surviving participants. No protocol or supporting text evidencing that no changes from original design. 85% compliance overall to circa 6 years follow up. |
| Steering Committee 1989 [32] | Yes | Yes | No | Yes | Yes | Yes | Low | Computer generated randomisation and calendar packs supplied centrally by Bristol Myers (1988 publication). Participants were not blinded to treatment but would not influence bleeding outcomes. Outcomes for >99.7%. 85.7% compliance in the aspirin group (14.2% taking aspirin in placebo). Separate Data Monitoring Group managed trial so assumed reporting agreed up front. 5 year follow up. |
| Ridker et al 2005 [40] | Don’t know | Don't know | Yes | Yes | Yes | Yes | Unclear | Design published in 1992 but not available online to check randomisation details. Baseline characteristics similar. >97% follow up and ITT used. 10 year trial. No info on % compliance but sensitivity analysis carried out on women who had taken less than 2/3 medication but no overall information on the % compliance. Follow up data censored at point of non-compliance |
| International Stroke Trial 1997 [35] | Don’t know | Don’t know | Yes | Don't know | Don't know | Don't know | Unclear | Randomisation method not stated. No baseline information provided. ITT used. Drop out rate 50%. No information on compliance during follow up (range 1-7 years). |
| SALT collab.group 1991 [34] | Yes | Yes | Yes | Yes | Yes | Yes | Low | Almost 100% follow up. 99% centres had compliance >90%. No formal protocol published but central coordinating centre monitoring trial. Median follow up 32 months. |

1. Many of the trials were published before internationally agreed criteria for randomised controlled trial reporting were in use. Where there was no evidenced sequence generation or allocation concealment but baseline values suggested no difference this was recorded in the notes section.

2. Blinding of subjects was not relevant to severe bleeding outcomes so did not lead to an 'unclear' score on overall assessment.

3. Complete outcomes. Stated % and scored Yes if ≥80%.

4. Included compliance with aspirin therapy and, if estimated as ≥80% across whole study period, scored 'yes'.
